# Praziquantel Reduces Maternal Mortality and Offspring Morbidity by Enhancing Anti-Helminthic Immune Responses

**DOI:** 10.3389/fimmu.2022.878029

**Published:** 2022-06-27

**Authors:** Matthew Lacorcia, Réka Kugyelka, Lorenz Spechtenhauser, Ulrich Fabien Prodjinotho, Youssef Hamway, Thomas Spangenberg, Clarissa Prazeres da Costa

**Affiliations:** ^1^ Technical University of Munich (TUM), School of Medicine, Institute for Med. Microbiology, Immunology and Hygiene, Munich, Germany; ^2^ Department of Biosciences, University of Salzburg, Salzburg, Austria; ^3^ Global Health Institute of Merck, Ares Trading S.A. (a subsidiary of Merck KGaA Darmstadt Germany), Eysins, Switzerland

**Keywords:** Schistosomiasis, transgenerational immune priming, Praziquantel (PZQ), anthelminthic, fetomaternal cross talk

## Abstract

Alongside the wide distribution throughout sub Saharan Africa of schistosomiasis, the morbidity associated with this chronic parasitic disease in endemic regions is often coupled with infection-driven immunomodulatory processes which modify inflammatory responses. Early life parasite exposure is theorized to drive immune tolerance towards cognate infection as well as bystander immune responses, beginning with *in utero* exposure to maternal infection. Considering that 40 million women of childbearing-age are at risk of infection worldwide, treatment with Praziquantel during pregnancy as currently recommended by WHO could have significant impact on disease outcomes in these populations. Here, we describe the effects of anthelminthic treatment on parasite-induced changes to fetomaternal cross talk in a murine model of maternal schistosomiasis. Praziquantel administration immediately prior to mating lead to clear re-awakening of maternal anti-parasite immune responses, with persistent maternal immune activation that included enhanced anti-schistosome cytokine responses. Clearance of parasites also improved capacity of dams to endure the additional pressure of pregnancy during infection. Maternal treatment also drove lasting functional alterations to immune system development of exposed offspring. Prenatal anthelminthic treatment skewed offspring immune responses towards parasite clearance and reduced morbidity during cognate infection. Maternal treatment also restored offspring protective IgE antibody responses directed against schistosome antigens, which were otherwise suppressed following exposure to untreated maternal infection. This was further associated with enhanced anti-schistosome cytokine responses from treatment-exposed offspring during infection. In the absence of cognate infection, exposed offspring further demonstrated imprinting across cellular populations. We provide further evidence that maternal treatment can restore a more normalized immune profile to such offspring exposed *in utero* to parasite infection, particularly in B cell populations, which may underlie improved responsiveness to cognate infection, and support the WHO recommendation of anthelminthic treatment during pregnancy.

## Introduction

Alongside the wide distribution throughout sub Saharan Africa of schistosomiasis, a chronic parasitic disease caused mainly by *Schistosoma mansoni* and *Schistosoma haeamatobium*, the morbidity associated with chronic infection remains characterized by immunomodulation and a hyporesponsiveness dependent on parasite burden (1). One potential driver of this phenomenon is *in utero* exposure to circulating schistosome antigens during maternal infection, which could influence the developing immune system of children, and modify anti-schistosome immunity. Immunomodulatory effects of schistosome infection are well-described in the context of bystander immune responses (1, 2), as are modulated immune responses following exposure to maternal infection (3–5), increased levels of schistosome adult-worm specific IgE and IgG in newborns cord blood whose mothers were infected during pregnancy (and not treated) (6). Schistosome antigens can drive immunomodulatory changes *via* presence in breastmilk (7), and have been detected as persisting in human children and murine offspring following exposure to maternal infection (8, 9). Murine models demonstrate that exposure to maternal infection with schistosomes consistently modifies immune development, with recent findings included impact upon B cell priming and DC-T cell interactions (5), as well as NKT cells (3).

Further infant outcomes of maternal parasite infection include earlier immune maturation (B cells), spontaneous (polyclonal) IgE, decreased infantile eczema episodes, anti-parasite IgG, lower vaccine-induced Ig-titres, suppressed T cell responses (anti-parasite and against vaccine antigens), altered T helper cell responses, increased regulatory responses (IL-10), and modified APC status (as reviewed in (10) and (11)). Considering that 40 million women of reproductive age are at risk of infection worldwide, treatment with Praziquantel (PZQ) during pregnancy could have significant impact on disease outcomes in these populations. PZQ has been effectively used for decades against adult schistosomes, *via* a recently described mode of action targeting a transient receptor potential melastatin ion channel (12). However, there is hesitancy to follow the WHO recommendation for treatment during pregnancy, as PZQ, marketed as Biltricide^®^, and donated for use within WHO-agreement as Cesol 600, is classed as a Category B drug for pregnancy, with animal studies not indicating a risk to the fetus, but with insufficient controlled studies in humans at this point (13). In existing studies, Praziquantel treatment has not been associated with adverse events regarding birth weight, fetal mortality, or congenital abnormalities (14). Anthelminthic strategies have been described to drive further changes associated with the re-awaking of the anti-parasite immune response, through disturbance of the regulatory states maintained by live parasites to ensure their survival within hosts (1). However, altered inflammatory outcomes have been associated with maternal treatment during pregnancy, including modified allergic parameters (15), and boosted immune responses to schistosome antigens after pregnancy (16). Enhanced immune priming during treatment is thought to occur due to death and degradation of worms, leading to increased load of worm antigens in the system, contributing to a natural partial-resistance hypothesized to develop of slowly as exposures to dead worms accumulate (17).

Treatment-induced shifts in schistosome-directed immune responses, particularly to antigens found in adult worms, can re-focus the tolerogenic modulations accumulated through chronic infection. Accordingly, treatment of *S. haematobium* infected children increased their IgG4 and IgE to adult worm antigen (AWA) as well as IgE to soluble egg antigen (SEA) (18), and a similar boost was seen following PZQ treatment of pregnant women infected with *S. mansoni*, where treatment caused a boost in IgG1, IgG2, IgG4 specific against worm antigens and enhanced IgE levels against SEA in the mothers (16), and enhanced cytokine levels (19). The well-established mouse model of *S. mansoni* [summarized in (20)], benefits from using a human pathogen in its preferred niche of the hepatoportal vasculature, and previously revealed materno-fetal effects including transcriptional changes in the placenta (21), later validated in human cohorts (6). Similarly, previous studies in murine systems of employing direct infection of previously schistosome-exposed offspring demonstrated transfer of schistosome antigens and anti-schistosome antibodies *in utero* and through breast milk, further associated with modified infections and reduced immune responsiveness (22), with detectable antibodies in neonatal mice arising from acute maternal infection further associated with reduced liver pathology and lower worm burden (9).

The shifting character of the anti-schistosome immune response across phases of infection is well described (23), with maternal infection phase, and likely associated cytokine profiles, further reflected in later offspring immune responses, able to differentially modify reactivity to allergens (21). Differences of priming effects also occur when comparing routes of maternal exposure, such as *via* breastmilk or *in utero* exposure (7). Parasite load presents another further variable, as-yet underexplored in maternal infection settings, yet determinants of worm burden are shown to impact host bystander immune responses (2). As such, the shared mechanisms behind transgenerational effects on cognate infection remain unclear, and with suggestions that these may involve processes linked to trained immunity (24) in innate cells, or specific priming in adaptive cells, such as in B cells, as has also been shown for other infections (25).

In line with these human cohort studies and murine models, here we focused on the interaction between immune imprinting effects of maternal helminth infection during pregnancy, and anthelminthic treatment with PZQ. Using this murine model, we explored the added implications of maternal treatment in transgenerational priming, and more specifically the hypothesis that the immune reactivation events triggered by PZQ-induced worm death during maternal treatment can imprint offspring immunity, with particular regard to cognate schistosome-direct immune responses. We link this to a profile of how prenatal treatment can disrupt the steady state immune priming imprinted *via* maternal parasite infection, changes that may not only underlie responses to schistosome infection, but also whether treatment of maternal infection can restore a more normalized immune profile to such offspring exposed *in utero* to parasite infection. These data provide supporting information regarding the WHO recommendation of anthelminthic treatment during pregnancy.

## Results

### Beneficial Impact of Anthelminthic (PZQ) on Maternal Schistosomiasis Is Accompanied by Persistent Maternal Immune Activation

**Figure 1:**
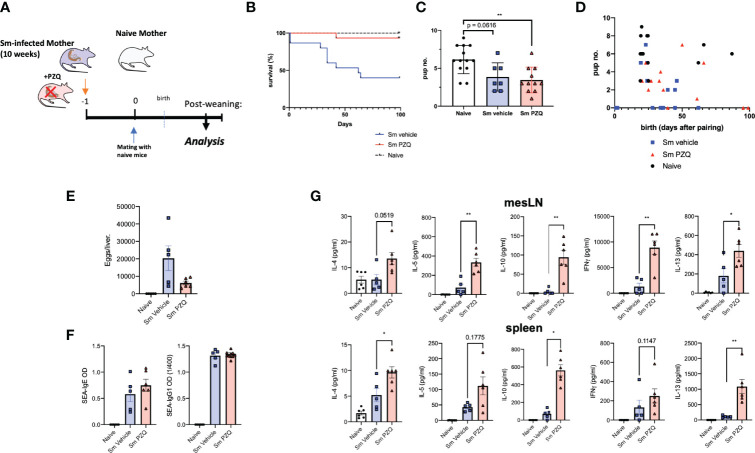
Impact of Anthelminthic (PZQ) on Maternal Schistosomiasis. **(A)** Model of exposure to maternal schistosomiasis, with (“Sm PZQ”) or without (“Sm vehicle”) additional anthelminthic treatment with PZQ in female mice, with subsequent maternal immune analysis post-weaning, with pairing occurring at day 0. **(B)** Survival curve of infected, treated, and naïve dams over the 100-day mating period post-anthelminthic treatment. **(C)** Number of pups per litter for dams with successful mating outcomes. **(D)** Comparison of pup number per litter with time (days) until birth. **(E)** Numbers of schistosome eggs per liver of infected dams. **(F)** SEA-specific IgE and IgG1 antibody titres in serum of dams. **(G)** Levels of secreted IL-4, IL-5, IFNγ, IL-10, and IL-13 as determined by ELISA in splenocyte and mesenteric lymph node cell culture supernatants after *ex vivo* re-stimulation with schistosome egg antigens (SEA). **(B)** through **(D)** is pooled from 2 independent maternal cohorts, with an initial total of 15 dams per condition. **(E)** through **(G)** show data from a representative maternal cohort, with an initial 7 dams per condition. Statistical differences induced by treatment between infected dam cohorts obtained *via* two-tailed *via* student’s T-test, or two-tailed Mann-Whitney U-test where data did not fit a normal distribution, *p < 0.05, **p < 0.01, shown as mean ± SEM.

In order to compare the transmaternal priming effects induced by prenatal anthelminthic treatment, maternal mice were divided into groups at week 10 of infection, following the type-2 dominant immune phase that peaks between weeks 7-9, at which time half of these were given a single dose of 400mg/kg PZQ, the other half only the vehicle (as per scheme in **Figure 1A**), and paired with naïve males. By the end usual 21-day gestation period, there was a clear difference in the successful survival of treated dams, compared to untreated dams, some of which needed to be removed from the experiment, unable to successfully bear the additional burdens of birth and the nursing period alongside a heavy parasite burden, as indicated by the drop in the blue line (**Figure 1B**). Although treatment clearly enhanced the ability of infected dams to effectively manage the requirements of pregnancy and birth, those dams who were infected had significantly smaller litter sizes compared to uninfected, naïve dams, regardless of subsequent treatment (**Figure 1C**). This was further reflected in the more regular gestation period of naïve mothers, in general giving birth in relative unison at the 3-week time point, compared to the more variable infection groups (**Figure 1D**). Upon end-point analysis, all PZQ-treated dams showed typical liver pathology of schistosomiasis upon visual inspection of successful patent infection, with the presence of eggs, although with visible trend of lower average egg number per liver compared to untreated infected dams (**Figure 1E**). Perfusion of livers yielded no surviving worms from treated mice, nor could these be identified in the intestines, as further confirmation of successful infection followed by successful treatment-based killing of the parasites. PZQ treatment did not appear to have a detectable effect on schistosome (egg)-specific antibody titers (**Figure 1F**).

Alongside these expected beneficial modified outcomes to infection, treatment of infection led to a lasting (re-)activation of immune responses in infected dams. Across mesenteric lymph nodes (mesLN) and spleens, *ex vivo* responses to schistosome egg antigens (SEA) demonstrated a clear immune enhancing effect of maternal treatment. This included enhanced levels of IL-4, IL-5, IFN-gamma, and IL-13 in supernatants of SEA-stimulated cell suspensions from either or both of spleen and mesLNs (**Figure 1G**), and indicates not only a degree of maternal immune activation (MIA) previously described for maternal cytokine alterations following viral infection (or its mimicry) during pregnancy (26), but a persistence of altered cellular functionality many weeks after treatment. TCR-based stimulation yielded similar enhanced responses from cells of treated mothers, with clearly increased IL-4, IL-10, and IL-13 responses from mesLN cells and splenocytes (**Supplement 1A**). Serum cytokine levels revealed long-term maintenance of altered profiles even in the case of treatment, with infection increasing IL-2, IL-13, GM-CSF, IL-5, among other cytokines, but these were not markedly altered following treatment (**Supplement 1B**).

### Prenatal Anthelminthic Treatment Skews Offspring Immune Responses Towards Parasite Clearance

**Figure 2:**
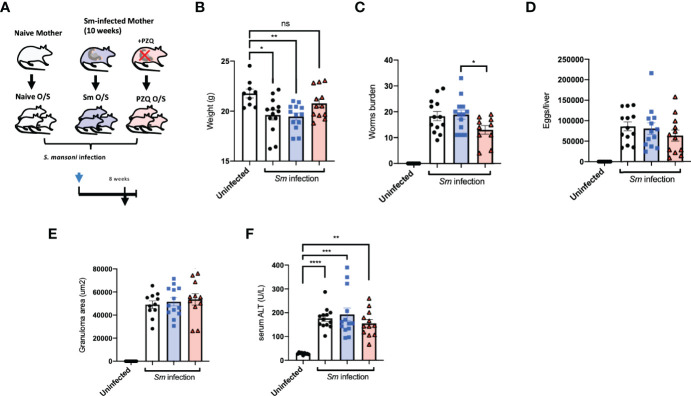
Prenatal anthelminthic treatment modifies infection outcomes during cognate schistosomiasis. **(A)** Generation of offspring exposed to maternal schistosomiasis, with (“PZQ O/S”) or without (“Sm O/S”) additional anthelminthic treatment other mothers, or from naïve dams (“Naïve O/S”), and subsequent cognate infection of all 3 groups with schistosomiasis. **(B)** Weight of adult offspring following 8-weeks of schistosomiasis. **(C)** Number of worms retrieved from infected offspring mice. **(D)** Extrapolated schistosome egg count per liver of infected offspring mice. **E)** Average size (area) of granulomas observable within livers of infected offspring mice after Masson’s trichrome stain of liver sections. **(F)** Alanine aminotransferase (ALT) levels in serum after cognate infection. Data from 2 independent infection experiments, each consisting of offspring from all 3 maternal exposures, with total n = 13-18 per infected group, with mothers: n = 3-6 per group. Statistical differences directly attributable to maternal treatment (i.e.: between offspring of infected dams) obtained *via* two-tailed *via* student’s T-test, or two-tailed Mann-Whitney U-test where data did not fit a normal distribution, or Kruskal-Wallis test plus subsequent individual comparisons from among all groups, *p < 0.05, **p < 0.01, ***p < 0.001, ****p < 0.0001, ns = not significant, shown as mean ± SEM.

Whether enhanced maternal anti-schistosomal immune responses induced by prenatal PZQ administration impacted subsequent offspring immunity to cognate infection was examined by *S. mansoni* infection of offspring either exposed to treated or untreated maternal schistosomiasis, as described earlier. These offspring themselves were examined after the 8^th^ week of infection (depicted in **Figure 2A**). A typical loss of bodyweight during infection was observed in naïve offspring as part of the disease progression during infection compared to uninfected controls. Comparing the effects of maternal anthelminthic treatment, those offspring of PZQ-treated infected mothers exhibited closer to normal bodyweight after patent infection, compared to the lowered weights of those exposed to untreated schistosomiasis (**Figure 2B**). This was further associated with a reduced worm burden in these PZQ-treatment exposed offspring compared to those born to infected, untreated mothers, showing on average 31% less worms (*P* = 0.0278), as retrieved from liver perfusion and further inspection of intestines (**Figure 2C**). Average eggs per liver showed similar trends, which although were not significantly different between offspring cohorts (**Figure 2D**), were significantly correlated to number of retrieved worms (**Supplement 2A**). Examination of liver sections from infection offspring cohorts did not reveal major differences in terms of granuloma formation in association with pre-exposure to maternal schistosomiasis (**Figure 2E**). Levels of alanine aminotransferase (ALT) in serum, as indicative of hepatic disease, weakly followed the pattern seen in worm burden, but did not reveal major differences between infected offspring cohorts (**Figure 2F**). Offspring serum cytokine levels during cognate infection further reflected the milder disease progression in offspring of PZQ-treated mothers compared to other groups, showing lower levels of infection-induced CCL5 (RANTES), and indications of lower amounts of IL-13, IL-5, IL-1b, IL-10, and IL-12p40 compared to standard infection of unexposed offspring (**Supplement 2B**).

### Prenatal Anthelminthic Treatment Enhances Schistosome-Specific Immune Responses

**Figure 3:**
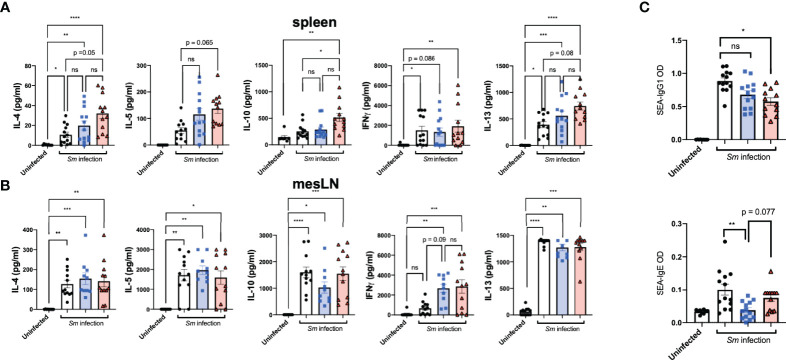
Prenatal anthelminthic treatment modifies schistosome-directed cellular and humoral immune responses following cognate schistosome infection of offspring. Levels of secreted IL-4, IL-5, IFNγ, IL-10, and IL-13 as determined by ELISA in splenocyte **(A)** and mesenteric lymph node **(B)** cell culture supernatants after *ex vivo* re-stimulation with schistosome egg antigens (SEA). **(C)** SEA-specific IgE and IgG1 antibody titres in serum of dams. Data from 2 independent infection experiments, each consisting of offspring from all 3 maternal exposures, with total n = 13-18 per infected group, with mothers: n = 3-6 per group. Statistical differences analysed *via* Kruskal-Wallis test plus subsequent individual comparisons from among all groups, *p < 0.05, **p < 0.01, ***p < 0.001, ****p < 0.0001, ns = not significant, shown as mean ± SEM.

*Ex vivo* re-stimulation with SEA of offspring-derived splenic and mesLN cell suspensions after infection revealed distinct priming effects resulting from maternal exposure in terms of subsequent cytokine levels in culture supernatants. Specifically, T_H_2-associated cytokines IL-4, IL-5, IL-10, and IL-13 from splenic cell preparations appeared elevated in offspring exposed to treated maternal schistosomiasis (**Figure 3A**). Further TCR-based stimulation induced increased levels of IL-13 production from PZQ-treatment exposed offspring during cognate infection (**Supplement 3**). In terms of site-specific effects, interestingly both treated and untreated pre-exposed offspring groups showed induction of IFN-gamma compared to uninfected mice in their mesLN suspensions, (**Figure 3B**) not observed in infected offspring from naïve mothers, indicating further disturbance of usual cytokine dynamics through prior transmaternal exposure to infection. This was not reflected in spleens, and more generally mesenteric cytokine responses (potentially due to proximity to the parasite niche) appeared to be a greater site of variation than spleens.

Antibody responses are induced strongly through schistosome infection, and as already described both schistosome-specific and polyclonal antibody titers have been suggested as induced through maternal helminth exposures. Overall, maternal schistosome infection drove lower SEA-IgG1 schistosome responses during infection (**Figure 3C**, upper), compared to infection of previously unexposed mice. Interestingly, however, was the apparent effect of maternal exposure on schistosome-specific IgE levels, known to be a specific part of the protective clearance response. Here, prior exposure to live maternal infection drove clearly lower levels of IgE (**Figure 3C**, lower), indicating a potential suppressive role on the ability to mount protective antibody responses (from live maternal infection), potentially indicating some degree of tolerance or ineffective response. This appeared instead restored with maternal PZQ-treatment, with those offspring instead displaying SEA-IgE titers closer to those of unexposed offspring.

### Prenatal Anthelminthic Treatment Redirects Transgenerational Priming of DC and B cell Compartments

**Figure 4:**
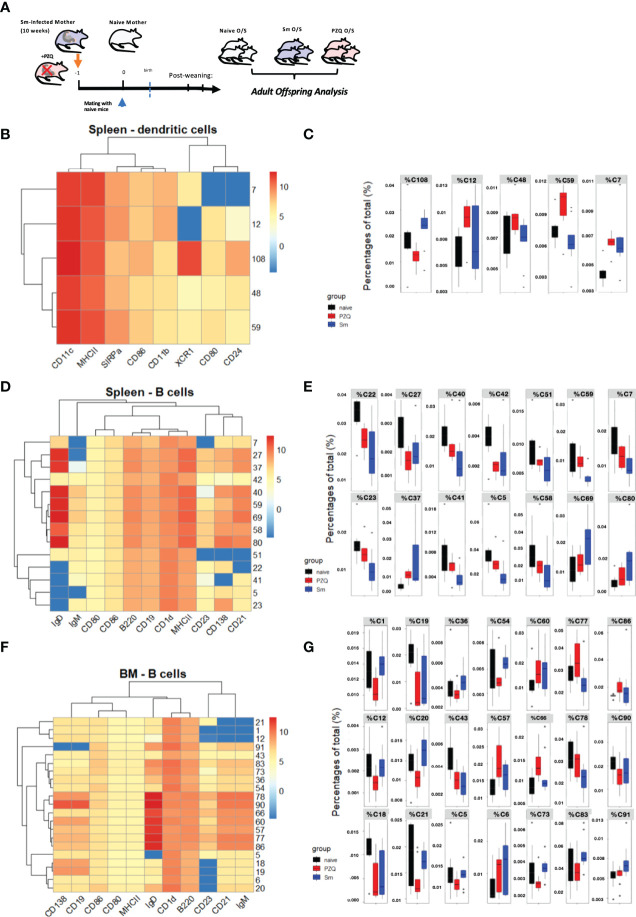
Prenatal anthelminthic treatment redirects transgenerational priming via maternal schistosomiasis. **(A)** Generation of adult steady-state offspring exposed to maternal schistosomiasis, with (“PZQ O/S”) or without (“Sm O/S”) additional anthelminthic treatment other mothers, or from naïve dams (“Naïve O/S”). FlowSOM- based splenic DC analysis, with **(B)** as corresponding heatmap of cluster identities and **(C)** showing differential enrichment of clusters (cluster % of total cells) quantified according to offspring groups. Differential clusters profiled in heatmap **(D)** of splenic B cell populations, and quantified per group in **(E)**. Differential clusters from bone marrow B cell populations profiled in heatmap **(F)** and relative cluster enrichment quantified per group in **(G)**. Data from 2 independent experiments, each consisting of offspring from all 3 maternal exposures, with total n = 6-10 per group, with mothers: n = 3-6 per group.

We then performed a further profiling of changes induced by maternal schistosomiasis, and in particularly how these are further impacted by the special case of PZQ treatment, within the steady-state (as in the overview **Figure 4A**). There were no clear alterations in serum cytokine concentrations, although some indications of mild increases in T_H_2-type cytokines (including IL-4 and IL-5) after exposure to maternal schistosomiasis (**Supplement 4A**), with only limited signs of impact from maternal PZQ-treatment, which also appeared not to discernably alter transmaternally-induced SEA-specific antibody levels nor shifts in total IgE or IgG1 antibody titers at steady state during this infection phase (**Supplement 4B**). We further conducted multiparametric cytometric analysis, and generation of relatively high-dimensional data, sampling spleens and bone marrow and multicolour antibody panels with a focus on dendritic cells and B cells, which we recently identified as site of maternal priming after schistosome infection (5). We applied the FlowSom algorithm (27, 28) to the generated flow cytometry-based data sets, generating self-organizing maps through unsupervised clustering of the acquired events, and developing those into minimal spanning trees (MSTs) that visualize and distinguish the cell populations within samples as based on identified clusters and metaclusters. The MST generated using manually pre-gated CD11c^+^MHCII^+^ conventional DCs (**Supplement 4D**), where group-wise comparisons identified 5 clusters where maternal schistosomiasis and/or its treatment with PZQ modified the relative enrichment of cells, the expression profile of these displayed in the heatmap (**Figure 4B**). Based on the proportion of cells within these clusters, as a percentage of total events (**Figure 4C**), both clusters C7 and C12 appear enriched in splenic DCs after exposure to maternal schistosomiasis, regardless of treatment, and are SIRP-α^+^XCR1^low^, indicative of cDC2-like subsets. C59, however, appears specifically enriched after exposure to PZQ-treated schistosomiasis, although its cluster-profile remains relatively indistinct. C108 has clear splenic cDC1 phenotype, high CD24 and XCR1, and relatively high CD86, and has clearly distinct group-wise effects: enriched after exposure to maternal schistosomiasis, but relatively reduced after exposure to treatment. Whether these cells represent the modified DC phenotype that we recently identified after chronic maternal schistosomiasis (5), particularly by bearing the CD86 signature of activation in both cDC1 and cDC2 populations *via* schistosomiasis and schistosome antigens (29), may warrant further mechanistic investigation in the switch that occurs following treatment.

When applied to pre-gated B cells, based on their B220 and/or CD19 expression, the FlowSOM output identifies many points of difference induced *via* maternal schistosomiasis (the expression profile of differentially enriched splenic B cell clusters shown in heatmap **Figure 4D**, with organization of all clusters instead visualized in the MST, **Supplement 4E**). In the spleen, untreated maternal schistosomiasis led to the largest effect in differential enrichment of the many clusters identified, with cells from offspring of PZQ-treated mothers instead showing an intermediate phenotype between naïve and schistosome-exposed (quantified in **Figure 4E**) with C59 representing a potentially interesting exception, with PZQ-primed offspring showing similar levels to naïve mice, and the underrepresentation of these MZ-like, CD1d^hi^ cells potentially indicating a tolerogenic immunomodulation imprinted by the live-infection alone. Additionally, both Sm-primed offspring groups shown underrepresentation of C27, which forms its own metacluster (MC8), and exhibits a CD21^lo^, IgM^hi^. IgD^lo^ transitional B-cell-like phenotype. In the bone marrow, however, numerous clusters were identified to be differentially enriched in schistosome-exposed compared PZQ-treatment exposed offspring (expression profiles of these shown in heatmap **Figure 4F**, cluster proportions quantified in **Figure 4G**, and full MST visualization of cluster organization shown in Suppment 4F). This includes a set of clusters that exhibited marginal zone-like or IgM^+^ innate-like memory phenotype (clusters 20, and 21, 1, and 12, respectively), which are all lowered in offspring by maternal PZQ-treated infection, with some instead showing enrichment after live schistosomiasis (such as cluster 20), which, considering the noted roles of these cells in maintenance of homeostasis and production of regulatory cytokines (and similarity to phenotypes displayed by SEA-induced regulatory B cells (29)), could be involved in the switch from tolerance to active protection observed in during cognate infection of offspring from these groups. Similarly, the clusters C91, C83, C73, C54, C20 show the highest amounts of CD1d, and further appear specifically enriched only after untreated Sm-only. These could further indicate cells with activation-induced regulatory functions as observed in schistosome infection, as an additional hallmark of such cells in previous studies was CD1d expression (30), which has also previously been linked to IL-10 production and therefore a pro-regulatory immune predisposition. Further, those B cell clusters significantly and specifically enriched in bone marrow of the PZQ-primed offspring (clusters C57, C66, C77 and C86), however, all fall within the central grouping of the heatmap, and are notable for high levels of IgD expression, as well as increased CD138, indicative together of an increased maturation profile, and therefore could be involved in the preparedness towards infection evidenced during cognate infection.

## Discussion

Environmental changes during early life are increasingly understood to impact proper development and education of the immune system. Of the many potential changes to the gestational environmental, maternal infection during pregnancy has been identified as a key factor driving heterologous immune responses (31). Maternal viral infection during pregnancy has been linked to numerous developmental changes, and has become explored experimentally through maternal immune activation models (26). Prenatal elimination of schistosomiasis through anthelminthic treatment has been shown to modify transgenerational effects of maternal schistosomiasis on atopy (15), and may poise the immune system for responsiveness to cognate infection. Indeed, recent discussions have highlighted the complexity of factors present during schistosome infection (11). Here, by means of a chronic schistosome infection mouse model, we uncover that the modified systemic immunological signature imparted by treating schistosomiasis is transferable from mothers to their offspring, distinguishable from active chronic infection by increased cytokine reactivity to schistosome antigens. Alongside improving pregnancy outcomes, prenatal treatment of schistosomiasis induced a form of maternal immune activation (otherwise described from maternal viral infection or models of this (26)) as signified by strongly increased antigen-specific T_H_1 as well as T_H_2 cytokines. We find that PZQ treatment boosted maternal cellular cytokine responses, in line with responses from treatment during pregnancy in human cohorts (19), and that this persists well beyond the point of weaning and therefore may impact the entire period of maternal contact for pups. The resultant improvements in offspring immunity to cognate schistosome infection indicate imprinting of this activation upon the developing immune system through transmaternal exposure. This altered offspring immune response by such treatment-exposed offspring to cognate schistosome infection, characterized by enhanced T_H_2-type cytokine responses to schistosome antigens, with a notably boost in SEA-induced IL-10 levels, and modified SEA-specific antibody levels. It is interesting to note that one of the clearest differences induced by exposure to treated maternal schistosomiasis was SEA-induced IL-10 levels, which reflects previous human studies where responses to schistosome antigens involving this cytokine was notable as elevated in children of PZQ-treated women (32). That maternal treatment was associated with normalized anti-schistosome IgE levels during cognate infection, otherwise suppressed by exposure to live maternal infection, reflects previous findings of detrimental effects of maternal schistosomiasis on effective induction of specific antibody titers (3). Treatment of helminth infection in humans was previously associated with increased IgG4 and IgE antibody responses (18), although pregnancy itself has been suggested to have a dampening effect of such boosting of antibodies (16). Recent data from our group demonstrated altered antibody transfer to children of schistosome-infected (yet untreated) mothers), in association with placental inflammatory parameters (6). Other infection parameters appeared largely unaltered by exposure to maternal infection in the absence of treatment, indicating only relatively faint specific immune effects in this setting, but the activating effect of maternal anthelminthic treatment was further associated with a modified appearance of infection-associated pathology, namely with reduced weight loss, lower worm burden, and associated signs of lower egg burden. Alongside the more general immune activation that follows treatment, the differential effect of exposure to PZQ treated versus non-treated maternal schistosomiasis may derive from specific induction on anti-worm versus anti-schistosome egg immune responses, the former not developing in the absence of worm death (17) and therefore may be enriched in the PZQ-treated groups. As with treatment leading to development of some resistance for infected hosts (33), this may similarly contribute to the transmaternally-primed partial resistance indicated in this study. This positions PZQ-treated maternal infection as a particular priming stimulus, that may differ in effect from untreated parasite infection, generally thought to downmodulate offspring anti-parasite responses to optimize the cross talk with the next generation of potential hosts, as observed in maternal malarial infection, where active (but not treated) maternal infection enhanced IL-10+ regulatory CD4+ T cells in cord blood that inhibited parasite-specific IFN-gamma responses (34). Additional dynamics of treatment (e.g.: PZQ given later during pregnancy) therefore represents a further avenue for research into this effect, as the timing of antigen release from dying worms, and the accompanying cytokine activation following treatment, could modify the amplitude of the priming effect on the next generation.

Despite the observance of reduced weight-loss during cognate infection following exposure to treated maternal schistosomiasis, the absence of a clear impact on schistosomiasis-induced liver pathology (in contrast to reports of total absence of egg-induced granulomas in some settings (9) suggests limits to protective effect of maternal treatment, at least in the context of a experimentally induced, high-burden infection. The reduced induction of serum cytokine levels, however, further supports an overall profile of reduced inflammatory pathology during infection. That these treatment-exposed offspring showed modified infection responses may further be linked to altered cellular priming, particularly within B cell activation state and B cell subset proportion, which we have observed after exposure to maternal schistosomiasis. Our cluster-based analysis allowed us to identify differences across subpopulations not immediately identified by manual gating, and further to assess these differences in an unsupervised way, and revealed the influence of maternal PZQ treatment. This includes a modification to the transgenerational priming driven by the parasites, which in the steady state did leave a signature of increased activation markers and modified enrichment of B cell subsets, evident in the bone marrow, which may further indicate priming alterations to hematopoiesis and trained immunity that remain to be elucidated, and deepens our profiling of the activation-induced enhanced regulatory phenotype described using manual gating-based analysis of DCs in our previous work (5). One such important change may be the underrepresentation of CD1d^hi^ B cells in offspring primed by live infection, with SEA-loaded cells previously shown to activate NKT cells in a CD1d-dependent manner (35) and NKT functionality recently shown to be affected by maternal schistosomiasis and associated with dampened responsiveness to immunization in a cascade involving instead lower IL-4 and reduced B cell priming, when evaluated early at the onset egg-deposition in patent infection in mothers (3). This indicates a potential pathway for transgenerational immune interference by maternal infection, with possible links to the modified regulatory network postulated to alter offspring bystander immune responses. Late into chronic infection these suppressed offspring bystander responses are instead associated with an IL-4-skewed, modified type 2 phenotype (5), typical of the regulatory network present during chronic infection itself (36). Further, detailed analysis of differences in antigen-specific immunity and repertoire changes may further illuminate the transmaternal priming effect driven by PZQ-treatment, including potential differences that may exist in effector T cell and antibody-responses against worm-derived antigens compared to those direct against egg antigens. Findings from human studies of treatment during pregnancy have found that although treatment during pregnancy augmented cytokine responses to SEA, that overall schistosome-directed cytokine responses were dampened during pregnancy (19), indicating further cross talk between infection and the tolerogenic immune processes characteristic of pregnancy. The timepoint at which maternal immune responses were measured during our current study was well after when such potential dampening effects of pregnancy would be likely to be observed. As such, a further opportunity offered by this murine model would be to explore in detail the direct effects on fetal development that occur during the *in utero* period (that is, earlier end-analysis of maternal immune responses), and deeper profiling of how infection and treatment alter the dynamic of cytokine environment to induce transgenerational priming following the maternal immune activation.

In humans, the currently running, prospective freeBILy study (registration number NCT03779437) gathers information on PZQ usage in pregnant women, a vulnerable population not usually included in mass drug administration programs, assessing the impact of diagnostics and PZQ-based treatment upon health-related quality of life in children, as well as incidence of schistosome infection in these individuals. The planned assessment of infant infection in association with maternal PZQ treatment (37) may add information about the priming effects of maternal infection and immune activation in human children. Through improved application of diagnostics, the results of such studies will facilitate integration of pregnant women into schistosomiasis treatment programs, as well as providing additional important safety information that will further inform guidelines (38). Our current murine-based study shows that maternal PZQ treatment can lead to non-specific priming effects, as well as partial resistance to cognate infection in mice, and would thus support the current WHO recommendation of anthelminthic treatment during pregnancy which is still met with hesitancy in the field. Further studies in human cohorts, such as FreeBILy, may provide additional valuable information on whether the observed effects translate to clinical settings.

## Materials and Methods

Animal experiments were performed under national and European Union guidelines 2010/63, and in accordance with specific approval by regional governmental authorities of Upper Bavaria (license AZ ROB-55.2-2532.Vet_02-17-145). C57BL/6J mice were purchased from Envigo (Germany). All mice were fed standard chow and housed under specific pathogen-free conditions at the Technical Univerity of Munich (TUM), Institute of Med. Microbiology, Immunology, and Hygiene, Munich, Germany.

C57BL/6 female mice were infected with 100 *S. mansoni* cercariae (using the in-house maintained Brazilian strain, with life cycle kept in mice), *via* subcutaneous injection into the flank (as demonstrated in **Figure 2C**, leading to on average 15-20 adult worms by week 8). These reached the 10^th^ week of infection (end of the peak T_H_2 immune response) when half of the infected mice (8 out of 16) were then treated with a single oral dose of PZQ at 400mg/kg in 10% Cremaphor (in H_2_O). This was given as a single treated at one-time point. Infected mice not receiving praziquantel received instead on the vehicle (10% Cremaphor), as did the control uninfected mice (all also *via* oral gavage). The following day, mice were mated with naïve male C57BL/6 mice. Offspring mice were weaned at 21-days of age. Upon end-analysis, dams were sacrificed, and successful infection plus treatment was confirmed by visual inspection of livers, in combination with absence of worms either retrieved *via* liver perfusion or inspection of intestines. For cognate infection experiments investigating offspring responses following maternal exposure, adult offspring cohorts (minimum of 6 weeks old) were infected *via* the same method, but were instead analyzed at week 8 after infection to assess peak responses. Infected mice were euthanized with sodium-pentobarbital (Narcoren, Boeringer Ingelheim) at a dose of 5ml/kg (800mg/kg) bodyweight.

For assessment of secreted cytokines in supernatant, cell suspensions were treated with SEA (produced in-house from hamster-derived schistosome eggs, at 20µg/ml), or anti-T cell receptor (TCR) stimulation *via* anti-CD3/CD28 (1µg/ml each). Unstimulated controls standardized to similar volumes using medium alone. Measurement of SEA-IgG1 and SEA-IgE in serum was performed with in-house ELISA as adapted from (39). Briefly, 1µg of SEA in 50µl PBS was used to coat each well of a 96-well microtiter plate overnight (NUNC). After washing 3x with PBST, these were blocked for 2h at RT with 3% (w/v) in PBS. After a further 3x washing, 100µl of serum diluted in BSA blocking buffer was probed for 2h at RT, washed again, then probed with 100µl detection antibody (biotinylated anti-mouse IgE or IgG1, Biolegend) diluted at 1:400 for 2h at RT. After washing, detection antibody was conjugated to 50µl Avidin-HRP peroxidase diluted 1:1000 for 30min at RT (in the dark), washed, then 50µl undiluted TMB substrate (eBioscience) was added per well and allowed to develop in the dark, before stopping with 50µl of 2 N H_2_SO_4_.

Granuloma area was calculated from formalin fixed (4%) paraffin-embedded cut sections (4µM) of liver and intestine samples after standard Masson’s trichrome staining and microscopic analysis as previously described (40) using up to 40 granuloma per mouse to calculate an average area µm^2^ per mouse. Determination of *S. mansoni* egg numbers per liver performed described previously (21) using 5% (w/v) KOH digestion of liver samples. Multiplexed analysis of serum cytokine levels was performed using Bio-Plex Pro Mouse Cytokine 23-plex Assay #M60009RDPD (Bio-Rad), according to manufacturer’s instructions with an initial 1:4 dilution of sample, and run on a Bio-Plex 200 System (Bio-Rad). Culture supernatants were instead analyzed using Mouse Ready-Set-Go ELISA kits (IL-4, IL-5, IFNγ)(eBioscience) or Mouse Duo-Set ELISA kit (IL-10)(R&D), according to manufacturer’s instructions. Alanine aminotransferase (ALT) levels in serum, indicative of hepatic disease, and were measured using a 1:4 dilution in PBS, and Reflotron GPT/ALT tests (Roche Diagnostics, Mannheim, Germany).

Flow-cytometry was performed as previously described (5), with cells were acquired using the flow cytometer Cytoflex S (Beckman Coulter) and initially analyzed in FlowJo 10 (v10 TreeStar). For B-cell analysis of spleen and bone marrow, live cells were gated on by excluding cells that were positive for propidium iodide (PI) (Biolegend), and pre-gated on cells positive for B220-APCH7 and CD19-BV510 (Biolegend). Further staining for B-cell subsets included IgM-BV605, IgD-PE-Cy7, CD23-AF700, CD21-PE-Dazzle594, CD138-PE, CD1d-BB700, CD80-FITC, and CD86-APC (all Biolegend), and MHCII-ef450 (eBioscience). For DC analysis, live cells were gated on by excluding cells that were positive for PI (double positive for PE and ECD), monocytes and monocyte-derived macrophages were excluded by expression of CD64 PerCP5.5 (Biolegend), Tissue resident Macrophages were excluded by the expression of F4/80 PE (eBioscience). cDC were identified by the high expression of CD11c-PE-Cy7 (Biolegend) and MHCII-ef450 (eBioscience). Further staining for DC subsets included XCR1-BV650, CD11b-BV510, CD172a (Sirp-α)-APC/Fire 750, CD24-AF700, CD80-FITC, and CD86-APC (all Biolegend).

For the FlowSOM analysis, fcs files were pre-gated in FlowJo for CD11c^+^MHCII^+^ conventional DCs or B cells based on B220 and/or CD19 expression. The exported samples were quality controlled (removal of low-quality events) using the PeacoQC algorithm (41) then processed with the FlowSOM workflow (28) and concatenated from individual samples. The SOMs were visualized using minimal spanning trees (MST). For dendritic cells, 145 922 cells were used for producing a self-organizing map (SOM) with a 11x11 grid, producing 121 clusters and 10 metaclusters (**Supplement 4D**). For splenic B cells, 1 559 562 cells were clustered in a 9x9 grid, resulting in 81 clusters and 12 metaclusters (**Supplement 4E**). From the bone marrow, 335 603 B cells were used in a 10x10 grid, producing 100 clusters and 12 metaclusters (**Supplement 4F**). To find clusters that were differentially represented in the experimental groups, median percentages of clusters of total number of cells and fold changes per cluster were compared using a Wilcoxon test. Significant clusters where characterized by the mean fluorescence intensities (MFI) of the markers used for clustering, heatmaps show scaled MFI values (pheatmap package https://github.com/raivokolde/pheatmap).

Statistical tests were performed using GraphPad Prism 8.0.2 for Windows (GraphPad Software, San Diego California USA). Data was subjected to D’agostino and Pearson omnibus normality test. Where specified, normalization was calculated using standard or z-values, where *Z = (x - mean of original experiment cohort)/standard deviation of original sample cohort*, which allowed re-scaling of data between repeat experiments, with normalized value calculated as: Z*(repeat experiment standard deviation) + mean of repeat experiment cohort, to minimize artefacts when pooling repeat experiments. Differences between sample sets with a normal distribution were analyzed using an unpaired, two-tailed Student’s t-test, while non-parametric data was assessed using an unpaired, two-tailed Mann-Whitney U-test, where p-values ≤ 0.05 were considered statistically significant. Analysis between multiple groups was performed using ANOVA or Kruskall-Wallis test for non-parametric data, with Dunn’s *post-hoc* test for comparison between individual groups. Pearson and Spearman correlations calculated using Rstudio Version 1.4.1106 (^©^ 2009-2021 RStudio, PBC).

## Data Availability Statement

The original contributions presented in the study are included in the article/**Supplementary Material**. Further inquiries can be directed to the corresponding author.

## Ethics Statement

The animal study was reviewed and approved by Animal experiments were performed under national and European Union guidelines 2010/63, and in accordance with specific approval by regional governmental authorities of Upper Bavaria (Regierung von Oberbayern, license AZ ROB-55.2-2532.Vet_02-17-145).

## Author Contributions

ML conceptualized the project, carried out the experiments, analyzed the data, produced the figures and wrote the manuscript. RK performed the experiments and contributed to experimental design, and conceptualized data pipelines, performed analysis, interpreted data and contributed figures and writing of the manuscript. LS performed experiments. UP performed an experiment and contributed to data processing. YH contributed to figure development and visualization, as well as the editing of the manuscript. TS conceptualized the study, supervised its execution and contributed to the discussion and editing of the manuscript. CdC conceptualized the study, supervised its execution including development of the manuscript and figures, and edited the manuscript. All authors contributed to the article and approved the submitted version.

## Funding

Financial support was provided by the Deutsche Forschungsgemeinschaft DFG CO 1469/16-1.

## Conflict of Interest

TS is an employee of Ares Trading SA, an affiliate of Merck KGaA, Darmstadt, Germany.

The remaining authors declare that the research was conducted in the absence of any commercial or financial relationships that could be construed as a potential conflict of interest.

The authors declare that this study received funding from Merck KGaA, Darmstadt, Germany. The funder was involved in the design of the study, data interpretation and preparation of the manuscript.

## Publisher’s Note

All claims expressed in this article are solely those of the authors and do not necessarily represent those of their affiliated organizations, or those of the publisher, the editors and the reviewers. Any product that may be evaluated in this article, or claim that may be made by its manufacturer, is not guaranteed or endorsed by the publisher.

## Supplementary Material

Supplement 1
**(A)** Levels of secreted IL-4, IL-5, IFNγ, IL-10, and IL-13 as determined by ELISA in splenocyte and mesenteric lymph node cell culture supernatants after TCR-based re-stimulation.

Supplement 2
**(A)** Correlation (spearman) between number of eggs per liver and worms retrieved *via* liver perfusion of infected offspring and inspection of intestines. **(B)** Selected readouts from Bioplex-based analysis of serum cytokines during cognate infection.

Supplement 3
**(A)** Levels of secreted IL-4, IL-5, IFNγ, IL-10, and IL-13 as determined by ELISA in splenocyte and mesenteric lymph node cell culture supernatants after TCR-based re-stimulation.

Supplement 4
**(A)** Bioplex-based analysis of serum cytokine levels in steady-state offspring groups, as analyzed *via* FlowSOM in. **(B)** SEA-specific IgE and IgG1 titers from the same offspring groups at steady-state, with **(C)** displaying total IgE and IgG1 antibody titers. Statistical differences analyzed *via* Kruskal-Wallis test plus subsequent individual comparisons from among all groups, *p < 0.05, **p < 0.01, shown as mean ± SEM. MST from Splenic DC analysis **(D)**, splenic B cell populations **(E)**, and bone marrow B cell populations **(F)**.
