# Supplementary figures and images for: Praziquantel Reduces Maternal Mortality and Offspring Morbidity by Enhancing Anti-Helminthic Immune Responses
Source: Front Immunol. 2022 Jun 27;13:878029. doi: 10.3389/fimmu.2022.878029 (PMC9272909; doi:10.3389/fimmu.2022.878029)

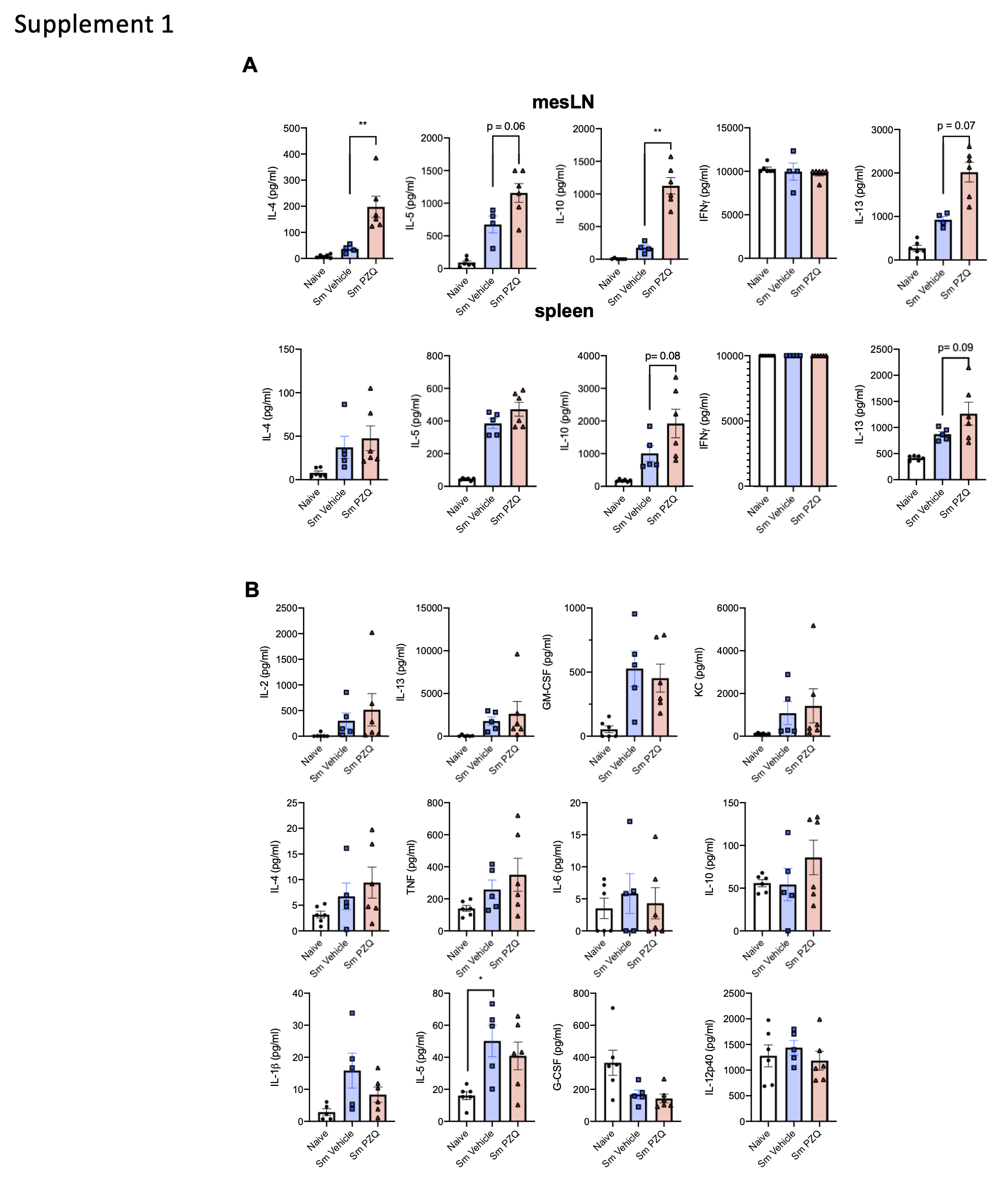

Supplement: Supplement 1 — (A) Levels of secreted IL-4, IL-5, IFNγ, IL-10, and IL-13 as determined by ELISA in splenocyte and mesenteric lymph node cell culture supernatants after TCR-based re-stimulation. [file Image_1.tiff]

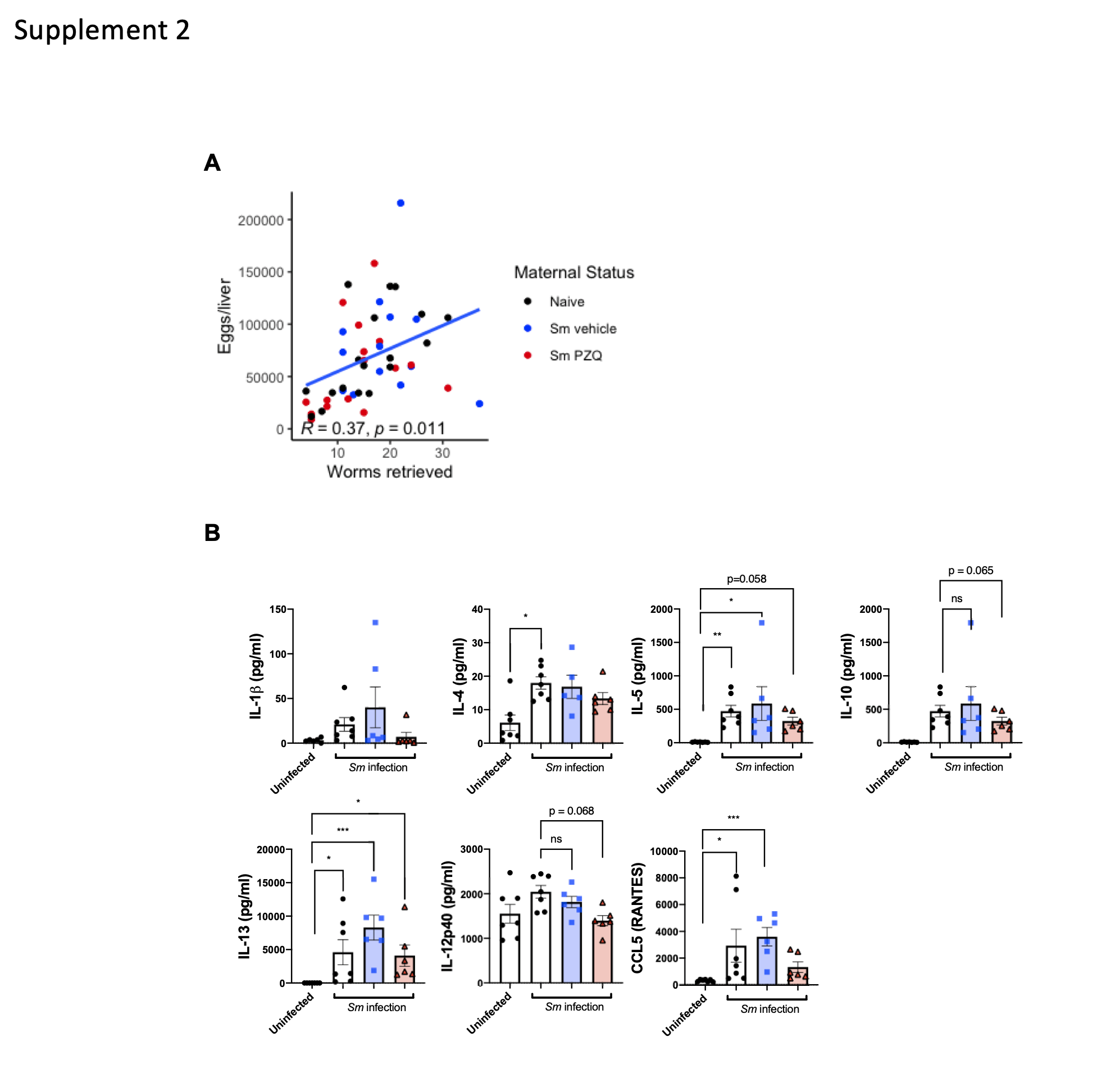

Supplement: Supplement 2 — (A) Correlation (spearman) between number of eggs per liver and worms retrieved via liver perfusion of infected offspring and inspection of intestines. (B) Selected readouts from Bioplex-based analysis of serum cytokines during cognate infection. [file Image_2.tiff]

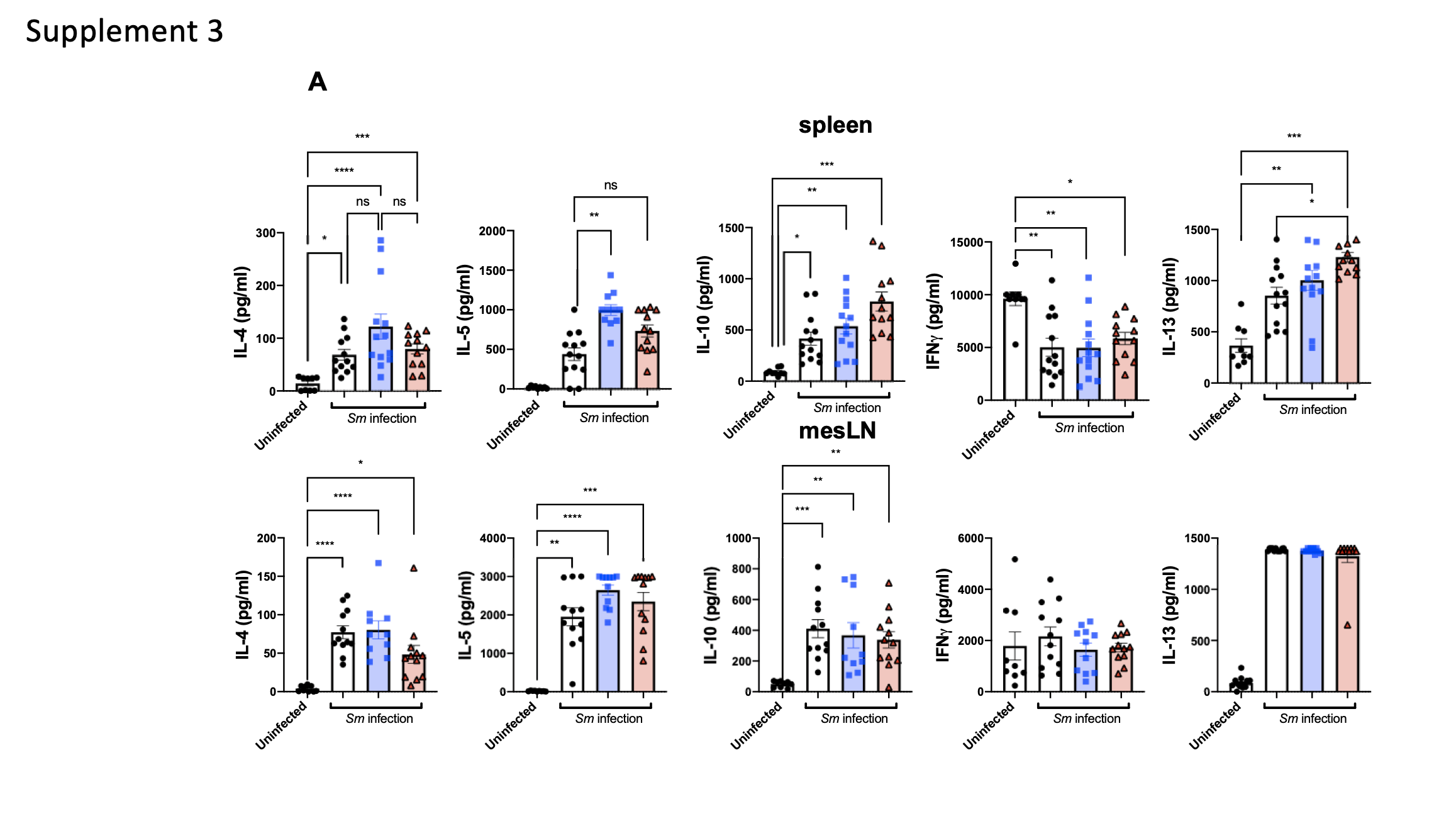

Supplement: Supplement 3 — (A) Levels of secreted IL-4, IL-5, IFNγ, IL-10, and IL-13 as determined by ELISA in splenocyte and mesenteric lymph node cell culture supernatants after TCR-based re-stimulation. [file Image_3.tiff]

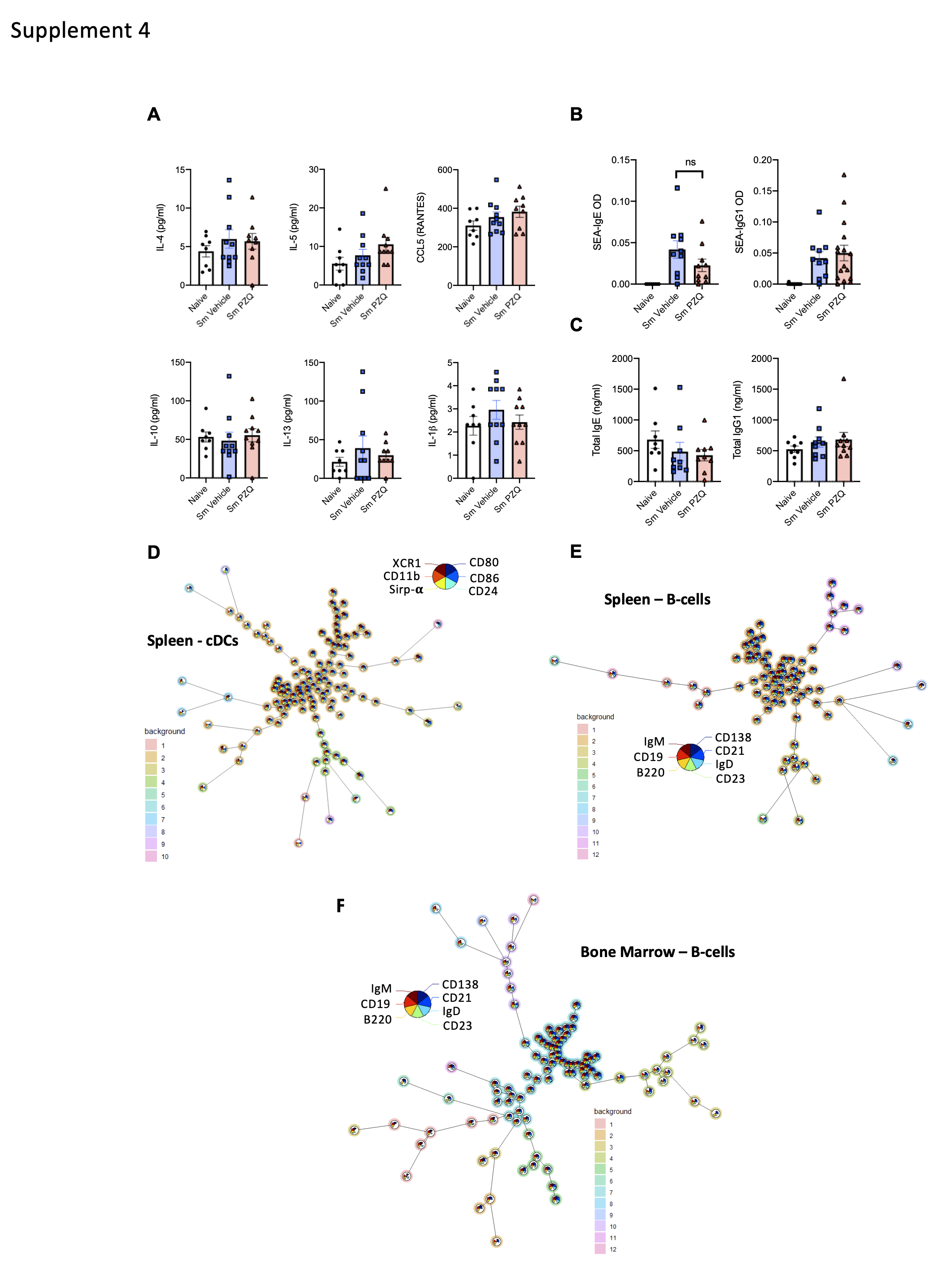

Supplement: Supplement 4 — (A) Bioplex-based analysis of serum cytokine levels in steady-state offspring groups, as analyzed via FlowSOM in. (B) SEA-specific IgE and IgG1 titers from the same offspring groups at steady-state, with (C) displaying total IgE and IgG1 antibody titers. Statistical differences analyzed via Kruskal-Wallis test plus subsequent individual comparisons from among all groups, *p < 0.05, **p < 0.01, shown as mean ± SEM. MST from Splenic DC analysis (D), splenic B cell populations (E), and bone marrow B cell populations (F). [file Image_4.tiff]
